# Repurposing sertraline sensitizes non–small cell lung cancer cells to erlotinib by inducing autophagy

**DOI:** 10.1172/jci.insight.98921

**Published:** 2018-06-07

**Authors:** Xingwu Jiang, Weiqiang Lu, Xiaoyang Shen, Quan Wang, Jing Lv, Mingyao Liu, Feixiong Cheng, Zhongming Zhao, Xiufeng Pang

**Affiliations:** 1 Shanghai Key Laboratory of Regulatory Biology, Institute of Biomedical Sciences and School of Life Sciences, East China Normal University, Shanghai, China.; 2 Department of Biomedical Informatics, Vanderbilt University School of Medicine, Nashville, Tennessee, USA.; 3 Institute of Biosciences and Technology, Department of Molecular and Cellular Medicine, Texas A&M University Health Science Center, Houston, Texas, USA.; 4 Center for Cancer Systems Biology and Department of Cancer Biology, Dana-Farber Cancer Institute, Harvard Medical School, Boston, Massachusetts, USA.; 5 Center for Complex Networks Research and Department of Physics, Northeastern University, Boston, Massachusetts, USA.; 6 Center for Precision Health, School of Biomedical Informatics, The University of Texas Health Science Center at Houston, Houston, Texas, USA.; 7 Department of Cancer Biology, Vanderbilt-Ingram Cancer Center, Vanderbilt University, Nashville, Tennessee, USA.

**Keywords:** Oncology, Therapeutics, Autophagy, Lung cancer

## Abstract

Lung cancer patients treated with tyrosine kinase inhibitors (TKIs) often develop resistance. More effective and safe therapeutic agents are urgently needed to overcome TKI resistance. Here, we propose a medical genetics–based approach to identify indications for over 1,000 US Food and Drug Administration–approved (FDA-approved) drugs with high accuracy. We identified a potentially novel indication for an approved antidepressant drug, sertraline, for the treatment of non–small cell lung cancer (NSCLC). We found that sertraline inhibits the viability of NSCLC cells and shows a synergy with erlotinib. Specifically, the cotreatment of sertraline and erlotinib effectively promotes autophagic flux in cells, as indicated by LC3-II accumulation and autolysosome formation. Mechanistic studies further reveal that dual treatment of sertraline and erlotinib reciprocally regulates the AMPK/mTOR pathway in NSCLC cells. The blockade of AMPK activation decreases the anticancer efficacy of either sertraline alone or the combination. Efficacy of this combination regimen is decreased by pharmacological inhibition of autophagy or genetic knockdown of *ATG5* or *Beclin 1*. Importantly, our results suggest that sertraline and erlotinib combination suppress tumor growth and prolong mouse survival in an orthotopic NSCLC mouse model (*P* = 0.0005). In summary, our medical genetics–based approach facilitates discovery of new anticancer indications for FDA-approved drugs for the treatment of NSCLC.

## Abstract 2

The antidepressant drug sertraline sensitizes non-small cell lung cancer cells to erlotinib through the induction of autophagy.

## Introduction

Recent advances in scientific, technological, and managerial efforts have been made to improve efficiency of drug discovery and development. However, the number of new the US Food and Drug Administration–approved (FDA-approved) drugs has decreased since 1950, and costs in drug discovery process has largely increased (1). Scientists in both academic and industrial fields have been seeking innovative technologies and approaches to decrease costs and augment efficiency in drug discovery. A potentially novel drug development technology, namely drug repurposing that screens existing drugs for new uses, draws great attention and delivers productivity (2–4). For example, thalidomide was repurposed by Celgene for the therapy of multiple myeloma (5). However, traditional methods for drug repurposing are still a time-intensive and costly endeavor. Developing innovative strategies with low costs and high efficiency, such as computational approaches, is urgently needed.

Cancer is a major public health problem in the world and is recognized as the second leading cause of death in the US (6). When compared with other types of cancer, lung cancer causes the most deaths in both males and females in the US. Lung cancer is characterized into 2 major histopathologic groups: non–small cell lung cancer (NSCLC) with ~80%–85% of cases and small cell lung cancer with ~13%–15% of cases (7, 8). Selective tyrosine kinase inhibitors (TKIs) show clinical efficacy and favorable toxicity profiles for the treatment of NSCLC as compared with traditional cytotoxic agents (9). However, NSCLC patients treated with TKIs often develop drug resistance due to acquired genetic and/or epigenetic events in individuals, and prolonged survival of patients is typically only a few months (10–12). Frequent genetic and epigenetic events have been observed in several key drivers, such as *EGFR*, *KRAS*, *ALK*, and *PIK3CA* (11). However, effective treatments for these actionable mutations remains insufficient. Therefore, repurposing FDA-approved agents with high efficacy and low toxic profiles is of great interest for the treatment of NSCLC (13–15).

The flood of large-scale data generated from electronic health records, parallel high-throughput sequencing, and genome-wide association studies (GWAS) has shown great impacts on current research (16–19). A recent study suggests that human genetic data generated from GWAS provides a valuable resource to select the best drug targets and indications in the development of new drugs, including anticancer drugs (20). Therefore, integrating large-scale medical genetics data through a computational approach provides great opportunities to identify new indications for approved drugs (21, 22).

In this study, we propose a medical genetics–based approach to find potential anticancer indications for FDA-approved drugs by integrating information from 2 comprehensive networks: the drug-gene interaction (DGI) and the gene-disease association network (GDN). Via this approach, we identify 2 FDA-approved antidepressant drugs (sertraline [trade name Zoloft] and fluphenazine) for a potentially novel anti-NSCLC indication. Specifically, our data provide various evidences that sertraline suppresses tumor growth and sensitizes NSCLC-resistance cells to erlotinib by enhancing cell autophagy. Our mechanism studies further reveal that the cotreatment of sertraline and erlotinib remarkably increases autophagic flux by targeting the AMPK/mTOR pathway. Notably, sertraline combined with erlotinib effectively suppresses tumor growth and prolongs mouse survival in an orthotopic NSCLC mouse model, offering a therapeutic strategy to treat NSCLC.

## Results

### A medical genetics–based approach for drug repurposing.

**Figure 1:**
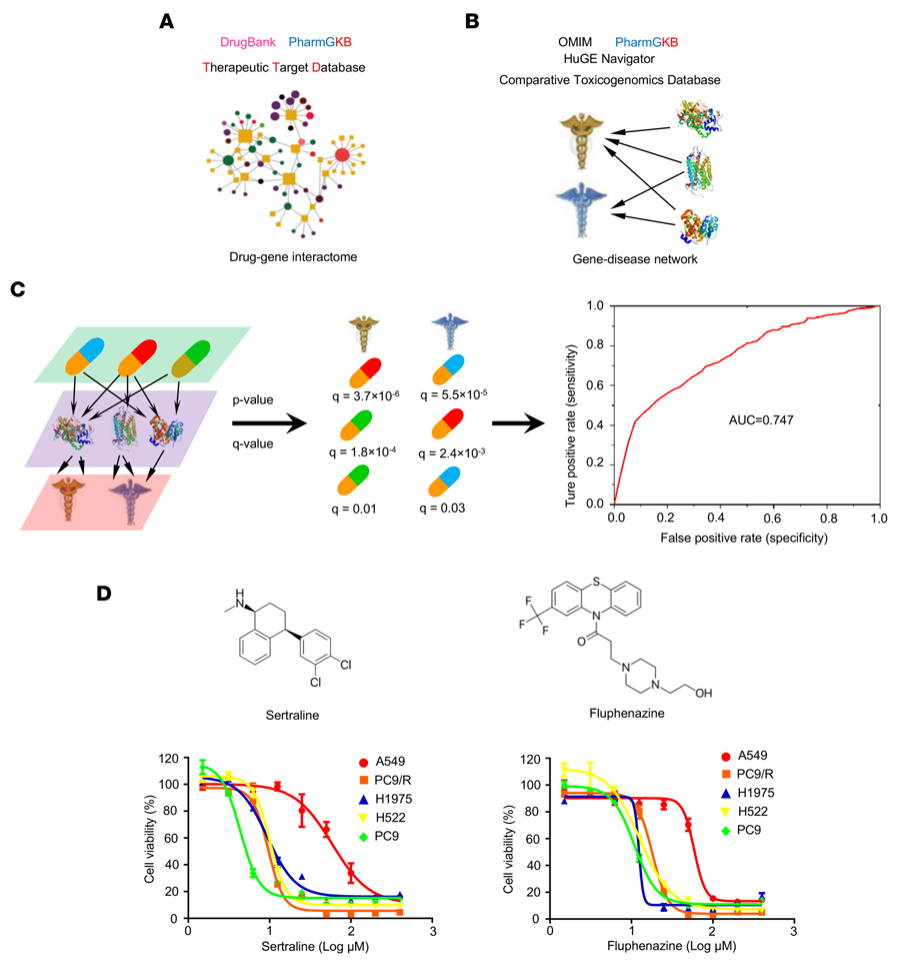
Diagram of medical genetics–based approach for drug repositioning. (**A**) A comprehensive drug-gene interactions (DGIs) was set up by integrating 3 public databases: DrugBank, PharmGKB, and Therapeutic Target Database. (**B**) A global disease-gene associations (DGAs) model was built by collecting data from 4 well-known data sources: the OMIM, HuGE Navigator, PharmGKB, and Comparative Toxicogenomics Database. (**C**) A new statistical model for predicting new indications for old drugs by integrating the DGIs and the DGAs. The performance of the medical genetics–based model was evaluated using a benchmark dataset. (**D**) The chemical structures and the dose-response curves of sertraline and fluphenazine in 5 representative NSCLC cell lines (A549, PC9, PC9/R, H1975, and H522) harboring different genetic characteristics. Cells were treated with a series of concentrations of sertraline or fluphenazine for 72 hours. The CellTiter 96 AQueous one solution cell proliferation kit was used to determine cell viability.

We developed a genetics-based approach to identify new potential indications for over 1,000 FDA-approved drugs. Specifically, we constructed a comprehensive DGI database by integrating the data from 3 public databases: DrugBank (v3.0; https://www.drugbank.ca/) (23), Therapeutic Target Database (TTD; https://db.idrblab.org/ttd/) (24), and PharmGKB database (https://www.pharmgkb.org/) (25). In DGIs, all drug target–coding genes were mapped and annotated using the Entrez IDs and official gene symbols from the NCBI database (26). All drugs were grouped using the Anatomical Therapeutic Chemical Classification System codes (www.whocc.no/atc/), which were downloaded from DrugBnak database (v3.0; ref. 23), and were further annotated using the Medical Subject Headings (MeSH) and unified medical language system (UMLS) vocabularies (27). Duplicated drug-gene pairs were removed. In total, we obtained 17,490 pairs connecting 4,059 FDA-approved or clinically investigational drugs with 2,746 targets (Figure 1A).

We next constructed a large-scale gene-disease associations (GDAs) database using the data from 4 public databases: the OMIM database (www.omim.org, December 2012) (28), HuGE Navigator (https://phgkb.cdc.gov/PHGKB/hNHome.action, December 2013) (29), PharmGKB (www.pharmgkb.org) (25), and Comparative Toxicogenomics Database (CTD, http://ctdbase.org/) (30). All disease terms were annotated using MeSH vocabularies (26), and the genes were annotated using the Entrez IDs and official gene symbols from the NCBI database (26). Duplicated pairs from different data sources were deleted. In total, we obtained 177,397 GDA pairs connecting 2,746 genes with 2,298 unique disease terms, which were further used to build a global GDA network (Figure 1B). Consequently, we combined the 17,490 drug-gene pairs with 177,397 GDA pairs to identify a set of genes that were targeted by a given drug and associated with a specific disease using a statistical framework (Figure 1C). We calculated the *P* values using the Fisher’s exact test and then adjusted the *P* values for multiple testing (*q* values) for each drug-disease pair using the Benjamini-Hochberg method (31). The hypothesis underlying this medical genetics–inference framework asserts that, if a set of genes that are targeted by a drug of interest is overrepresented in a given disease, this drug will have the high probability of a new indication for this disease based on the system pharmacology framework (9).

To evaluate our model performance, we collected a benchmark drug-disease association set comprising 1,593 pairs from the PharmGKB database (25). We computationally identified a total of 6,422 drug-disease association pairs and further evaluated computational performance following a strategy of 5-fold cross-validation. The area under the receiver operating characteristic (ROC) curve (AUC) was calculated, and our result showed that the AUC was 0.747, indicating that our computational model performed well (Figure 1C). We then chose NSCLC as a disease indication, and by setting *P* < 0.001 as a cutoff, we identified 95 clinical drugs showing predicted effects on NSCLC from over 1,000 FDA-approved drugs. Among these 95 predicted ones, 2 clinical antidepressant drugs, fluphenazine and sertraline, were in the top rank. Their *P* values were 2.4 × 10^–5^ and 1.2 × 10^–4^, respectively, suggesting their great potential to treat NSCLC. A recent study suggested that tricyclic antidepressants induced apoptosis in small cell lung cancer (15). However, the mechanism-of-action of sertraline and fluphenazine, tricyclic antidepressants harboring different chemical structures, in the suppression of NSCLC remains unknown (15).

We tested the antiproliferative activities of fluphenazine and sertraline in 5 representative NSCLC cell lines harboring mutations in *EGFR* or not: H522 (*EGFR* WT), A549 (*EGFR* WT; *KRAS* mutation), H1975 (*EGFR* T790M mutation), PC9 (*EGFR* 19-bp deletion in exon 19), and PC9/R (Figure 1D). The PC9/R cell line was generated by continuously exposing parental erlotinib-sensitive PC9 cells to increasing concentrations of erlotinib for a period of 6 months (32, 33). We found that sertraline and fluphenazine were cytotoxic to all tested NSCLC cells at micromolar (μM) range (Figure 1D). Particularly, the cytotoxicity of sertraline (IC_50_ = 4.40 μM in PC9 cells, IC_50_ = 11.10 μM in A549 cells, IC_50_ = 10.50 μM in H522 cells, IC_50_ = 9.40 μM in H1975 cells, and IC_50_ = 9.60 μM in PC9/R cells) was more potent than that of fluphenazine (IC_50_ = 10.90 μM in PC9 cells, IC_50_ = 58.92 μM in A549 cells, IC_50_ = 12.67 μM in H522 cells, IC_50_ = 12.36 μM in H1975 cells, and IC_50_ = 8.08 μM in PC9/R cells). Thus, we chose sertraline for further experimental validation.

### Sertraline and erlotinib combination reduces cell viability in NSCLC.

**Figure 2:**
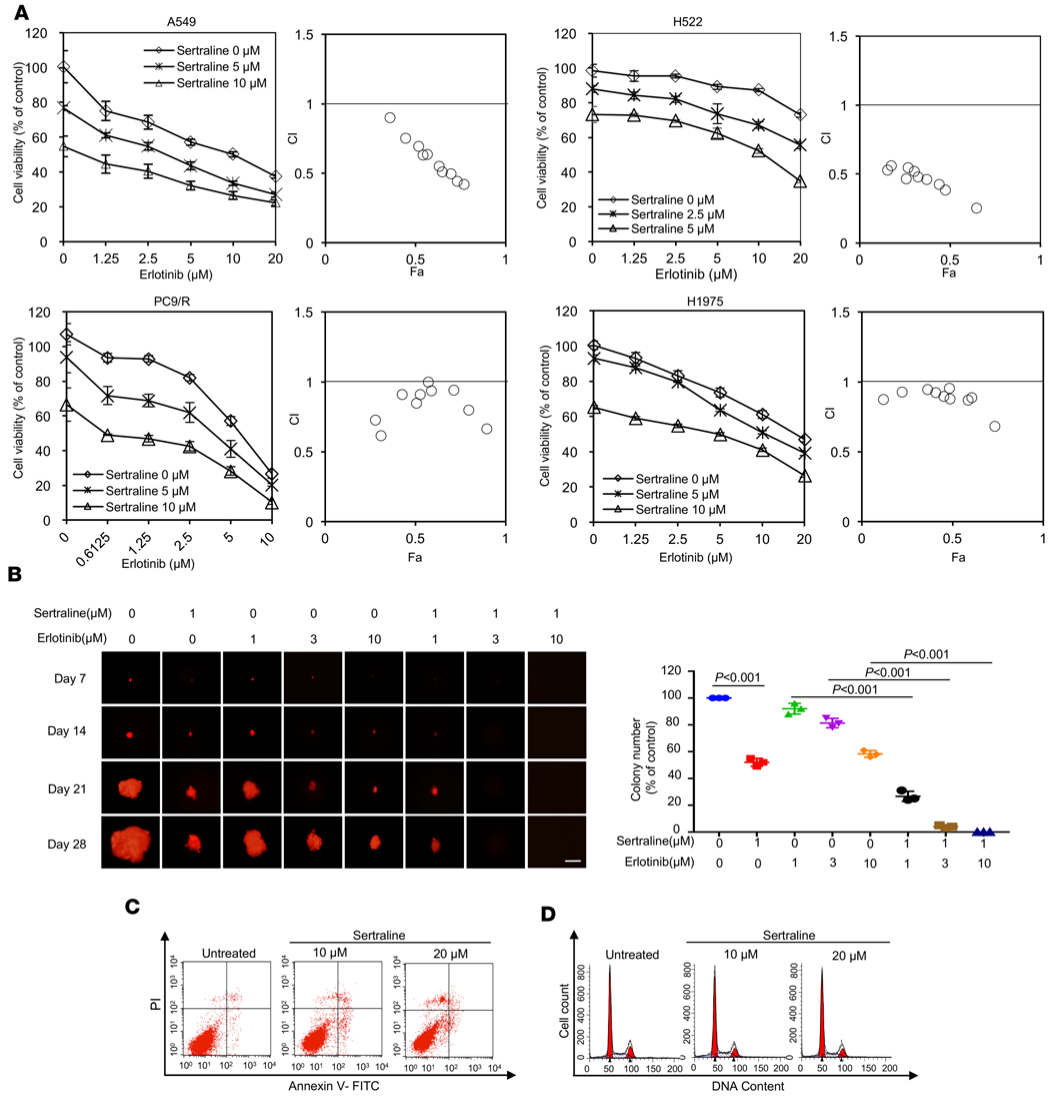
Synergistic effects of sertraline and erlotinib combination. (**A**) Sertraline alone or in combination with erlotinib decreased the growth of EGFR TKI–resistant NSCLC cells (A549, H522, PC9/R, and H1975) in vitro. Cells were treated with the indicated concentrations of sertraline, erlotinib, or sertraline plus erlotinib for 48 hours. Cell viability was measured by using the CellTiter-Glo luminescent cell viability kit, and combination index (CI) values were calculated using the Chou-Talalay equation. The data was presented by the fraction affected by the dose–CI (Fa-CI) plot. The Fa and CI values of 2 drugs at their combination of IC_50_ were listed in *x* axis and *y* axis (*n* = 3). CI values <1, =1, and >1 represent synergism, additive, and antagonism, respectively. (**B**) Representative images of fluorescent colonies. A549-mCherry (1,000/well in 6-well plates) cells were cultured in soft agar in the presence of sertraline, erlotinib, or combination drugs for 28 days. The colony growth was recorded every week using fluorescence microscope. Medium was changed every 3 days. Colonies (>100 μm in diameter) were counted. Scale bars: 1 mm. Data presented as mean ± SD (*n* = 3). *P* values were analyzed by 1-way ANOVA followed by Tukey’s multiple comparison test (set at 5%). (**C**) Percentage of apoptotic cells was determined by Annexin V and propidium iodide (PI) staining after sertraline treatment for 48 hours in A549 cells. (**D**) A549 cells were treated with sertraline for 48 hours; then, the cell cycle distribution was analyzed by flow cytometry using propidium iodide staining. All experiments were performed independently in triplicate. Error bars represent ± SD. Statistical significance level was set by *P* < 0.05.

EGFR TKIs are widely used as targeted agents for the treatment of NSCLC in clinical settings. However, most patients with *EGFR*-mutant lung cancer eventually develop acquired resistance to EGFR TKIs. Therefore, we examined the antiproliferative activity of sertraline in combination with erlotinib, an EGFR TKI, in the above NSCLC cell lines. As shown in Supplemental Table 1 (supplemental material available online with this article; https://doi.org/10.1172/jci.insight.98921DS1), PC9 was sensitive to erlotinib, while PC9/R, A549, H522, and H1975 cells were resistant to erlotinib, consistent with previous studies (32, 33). Interestingly, the combination of sertraline and erlotinib was more effective to inhibit cell viability than either single agent alone (Figure 2A). To investigate whether sertraline and erlotinib were synergistic, we calculated the combination index (CI) using CalcuSyn software (Version 2; Biosoft) (34). Our data showed that combining sertraline with erlotinib displayed a synergistically antiproliferative effect on A549, H522, PC9/R, and H1975 cells, and the CI values at different drug concentrations were all less than 1 (Figure 2A). Similar results were obtained by the CellTiter 96 AQueous one solution cell proliferation assays in the same cell lines (Supplemental Figure 1). To test whether the drug pair of sertraline and erlotinib in the suppression of cell growth had a general impact on proliferating cells, we performed the drug combination assays in the normal human lung fibroblast cell line MRC5, while we found that sertraline and erlotinib have no synergic effect on MRC5 (Supplemental Figure 2). A prolonged 3-dimensional (3-D) colony formation assay was also carried out to investigate whether the combined treatment of sertraline and erlotinib could cause irreversible growth arrest. We found that the drug pair produced a strongly synergistic effect to inhibit the colony formation of mCherry-tagged A549 cells (Figure 2B). To understand the antitumor mechanism of sertraline, we then investigate the effect of sertraline on apoptosis and cell cycle of NSCLC cells. As shown in Figure 2C, sertraline could not trigger obvious apoptotic cell death in A549 cells. In addition, caspase-3 target protein poly(ADP-ribose) polymerase (PARP) was not cleaved by sertraline or combination treatment (Supplemental Figure 3A). Consistently, the pan-caspase inhibitor Z-VAD-FMK failed to impair the cell killing triggered by either sertraline alone or in combination with erlotinib in A549 and H522 cells (Supplemental Figure 3B). These results suggest that sertraline does not induce caspase-mediated apoptosis, which was consistent with the previous finding (35). Meanwhile, sertraline has no effect on cell cycle, either (Figure 2D). Together, these results indicate that sertraline potentiates the anticancer effects of erlotinib in EGFR TKI–resistant NSCLC cells through induction of nonapoptotic cell death.

### Combining sertraline and erlotinib induces LC3-II accumulation in NSCLC cells.

**Figure 3:**
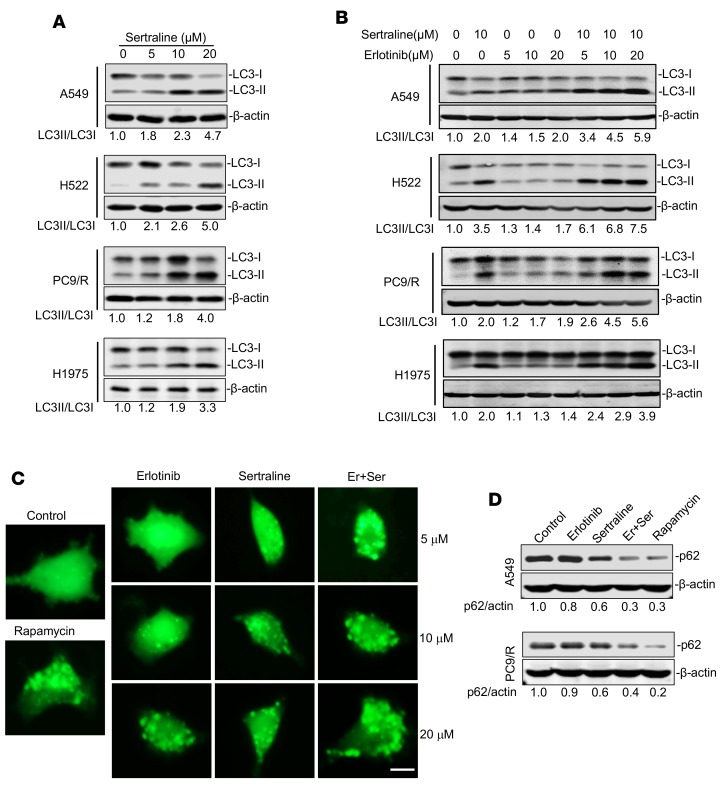
Drug combination of sertraline and erlotinib induces autophagy. (**A**) Sertraline increased the protein level of LC3-II in EGFR TKI–resistant NSCLC cell lines. A549, H522, PC9/R, and H1975 cells were treated with sertraline for 24 hours. The cell lysates were subjected to immunoblotting with indicated antibodies. (**B**) Sertraline in combination with erlotinib increased the level of LC3-II in EGFR TKI–resistant NSCLC cell lines. A549, H522, PC9/R, and H1975 cells were treated with sertraline, erlotinib, or drug combination for 24 hours. The cell lysates were subjected to sodium dodecyl sulfate polyacrylamide gel electrophoresis, followed by immunoblotting with indicated antibodies. (**C**) Increased GFP-LC3 puncta by different treatments. The representative images of GFP-LC3 puncta in A549 cells treated with vehicle, erlotinib, sertraline, or drug combination. Rapamycin (400 nM) served as the positive control. Scale bars: 20 µm. (**D**) Sertraline in combination with erlotinib downregulated intracellular expression of p62 in A549 and PC9/R cells. All experiments were performed independently in triplicate.

Previous studies showed that erlotinib could induce autophagy at clinically relevant concentrations in NSCLC cells (36). Our data also showed that sertraline augmented the anticancer effects of erlotinib by induction of nonapoptotic cell death. Therefore, we investigated whether the combination of sertraline and erlotinib induced autophagy that further contributed to the regression of NSCLC growth. We examined the formation of LC3-II, a key biomarker of autophagy (37), in 4 TKI-resistant NSCLC cell lines using Western blotting assays. As shown in Figure 3A, sertraline induced autophagy in a concentration-dependent manner in A549, H522, PC9/R, and H1975 cells. When combining with erlotinib, sertraline remarkably led to the accumulation of LC3-II (Figure 3B). Among them, A549 was more vulnerable to the combined therapy. We further investigated the generation of GFP-LC3-II puncta using a fluorescence microscopy. The results showed that sertraline in combination with erlotinib produced a significant induction of autophagy, and the number of cells with GFP-LC3 puncta was remarkably increased (Figure 3C). A previous study suggested that the intracellular level of p62 could serve as a marker of autophagic flux (38). We additionally found that p62 abundance was dramatically decreased by either sertraline alone or in combination with erlotinib in A549 cells and PC9/R cells (Figure 3D and Supplemental Figure 4), further confirming the induction of autophagy by the sertraline-containing treatments. These results indicate that sertraline combined with erlotinib coordinately induces autophagy in EGFR TKI–resistant NSCLC cells.

### Sertraline and erlotinib combination promotes autophagic flux in NSCLC cells.

**Figure 4:**
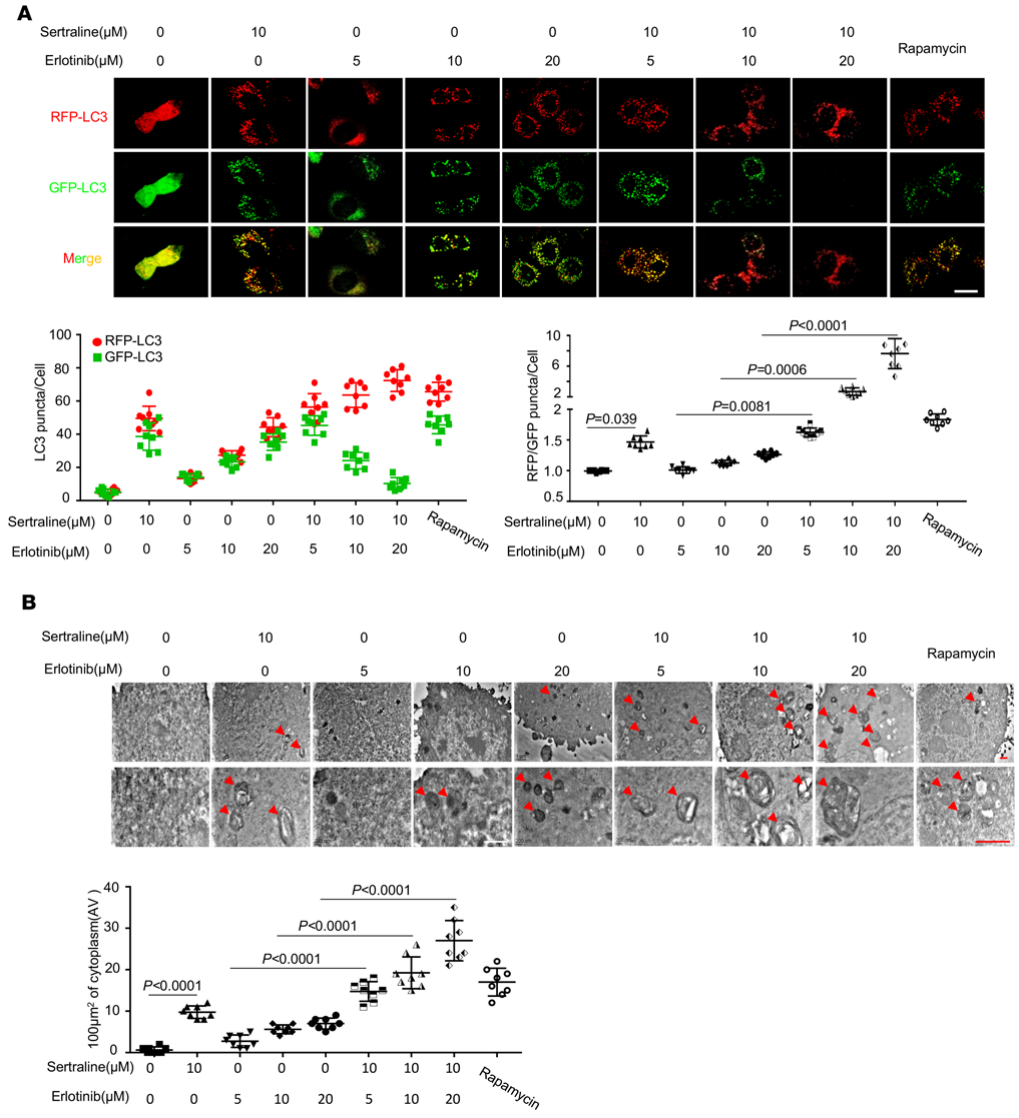
Sertraline, erlotinib, or their combination elevate autophagic flux in cells. (**A**) Elevated autophagic flux by different treatments. A549 cells were transfected with mRFP-EGFP-LC3 and treated with sertraline, erlotinib, or their combination for 24 hours. Confocal images showed autophagosome (mRFP-positive plus EGFP-positive) and autolysosome (mRFP-positive only) formation in cells. Rapamycin (200 nM) served as the positive control. Scale bars: 20 μm. (**B**) Combining sertraline with erlotinib induced the increase of autophagic vacuoles in A549 cells. The red arrows show autophagic vacuoles in treated cells. Scale bars: 1 μm. All data in **A** and **B** were presented as mean ± SD (*n* = 8). *P* values were analyzed by 1-way ANOVA followed by Tukey’s multiple comparison test, and *P* < 0.05 was considered statistically significant.

Tandem fluorescent protein–tagged LC3 is a useful marker for monitoring autophagic flux due to differential sensitivity of GFP and RFP fluorescent proteins to pH. We further utilized monomeric red fluorescent protein (mRFP) and EGFP tandem-tagged probes (mRFP-EGFP-LC3) to examine functional autophagy in A549 cells. EGFP easily loses fluorescence due to lysosomal acidity, while mRFP shows resistance to proteolytic degradation and maintains red fluorescence in autolysosomes (39). As shown in Figure 4A, A549 cells treated with sertraline had more mRFP-positive–only vesicles than mRFP/EGFP-positive ones compared with untreated cells, showing that sertraline treatment led to EGFP proteolysis in autolysosomes. Notably, the dual treatments of sertraline and erlotinib produced a much stronger mRFP fluorescence signal compared with either single agent alone (Figure 4A). These results indicated that sertraline and erlotinib cooperatively boosted functional autophagy. Transmission electron microscopy is a powerful tool to detect autophagic vesicles and autophagic flux in cells (40). We next used this method to examine autophagic flux in cells. Our results showed that treatments of sertraline or erlotinib alone led to an increased number of autolysosomes; however, this effect was largely augmented by the combination treatment (Figure 4B). Collectively, our results suggest that either sertraline alone or in combination with erlotinib could induce remarkable autophagic flux in EGFR TKI–resistant NSCLC cells.

### Autophagy induced by sertraline and erlotinib combination contributes to their cytotoxicity in NSCLC.

**Figure 5:**
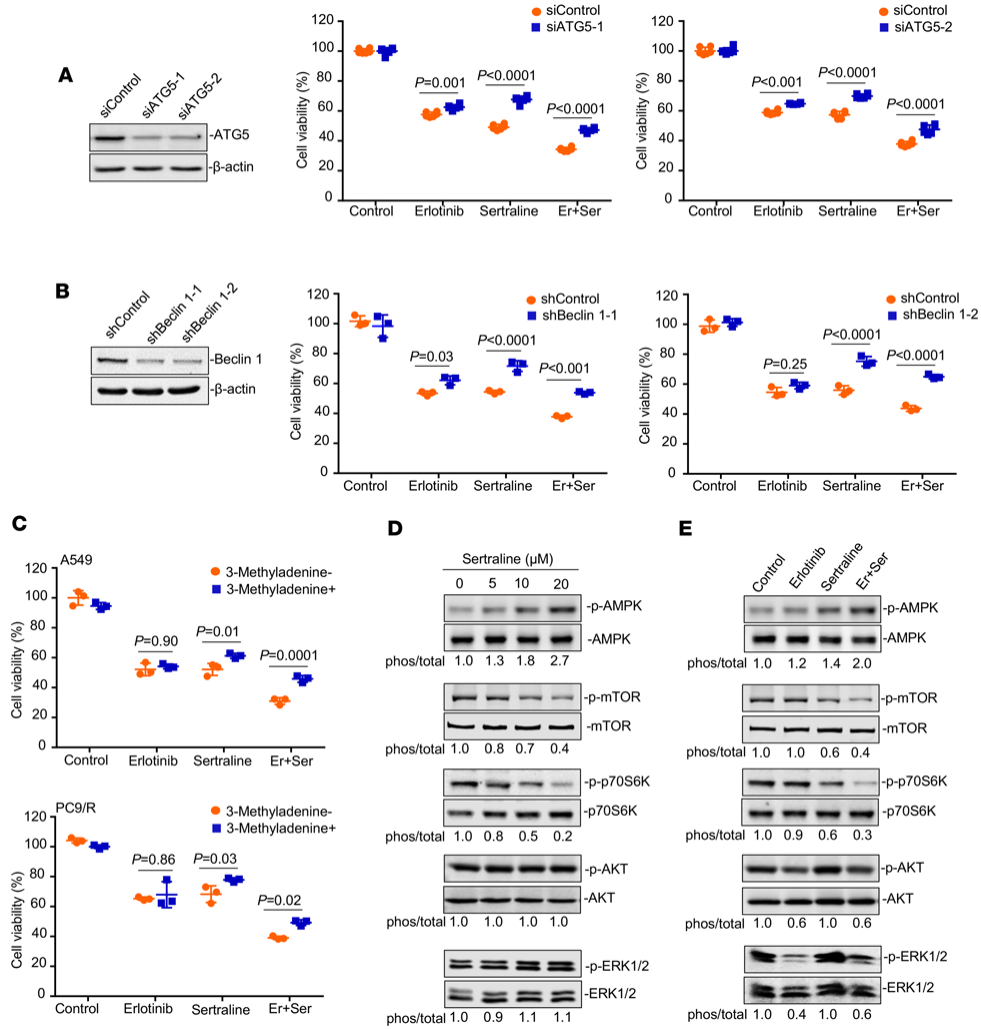
Sertraline alone and in combination with erlotinib induces autophagy through reciprocally regulating the AMPK/mTOR pathway. (**A** and **B**) Silence of ATG5 and Beclin1 significantly impaired the anticancer effect of sertraline or sertraline/erlotinib. A549 cells transiently transfected with siControl/shControl or siATG5/shBeclin1 were treated with erlotinib (10 μM), sertraline (10 μM), or drug combination for 48 hours (*n* = 3). Immunoblotting was used to determine the efficiency of the knockdown. (**C**) Pharmacological blockade of autophagy by 3-Methyladenine (3-MA) significantly inhibited the antitumor activity of sertraline or the drug pair in A549 and PC9/R cells. Cells were pretreated with 3-MA (1 mM) for 6 hours, followed by the treatments of erlotinib (10 μM), sertraline (10 μM), or drug combination for 48 hours. All data was represented as mean ± SD (*n* = 3). (**D** and **E**) A549 cells were treated with various concentrations of sertraline (5, 10, and 20 μM) or combination for 24 hours. The phosphorylation and basal levels of several key regulators of autophagy were probed by Western blotting. Quantification of relative density was shown. All experiments were performed independently in triplicate. *P* values in (**A–C**) were analyzed by 2-way ANOVA, followed Sidak’s multiple comparisons test, and *P* < 0.05 was considered statistically significant. Er, erlotinib; Ser, sertraline.

We next examined whether the elevated autophagic flux contributed to the impaired growth of EGFR TKI–resistant NSCLC cells triggered by the sertraline-containing treatments. We employed genetic knockdown of autophagy regulatory genes or pharmacological inhibitors of autophagy to validate the above effects. Our results showed that siRNA-mediated silence of *ATG5* significantly impaired the anticancer effect of the drug pair in A549 cells (Figure 5A). In addition, shRNA-mediated downregulation of *Beclin 1* also decreased the cytotoxic potency of the drug combination (Figure 5B). Furthermore, pharmacological blocking of autophagy by 3 different kinds of small-molecule inhibitors — 3-methyladenine (3-MA), chloroquine, and bafilomycin A1 — decreased the cytotoxicity of sertraline or its combination with erlotinib in A549 and PC9/R cells (Figure 5C and Supplemental Figure 5). These data suggest that increased caspase-independent autophagic cell death is largely involved in the mechanism-of-action of the combined therapy of sertraline and erlotinib.

### Sertraline induces autophagy by targeting the AMPK/mTOR/S6K signaling pathway.

To explore the potential mechanism-of-action underlying increased autophagy in response to combination therapy, we next examined the effect of sertraline and its combination with erlotinib on well-known autophagy regulators. As reported, the products of autophagy-related genes are regulated by nutrient and energy, in which the mTOR and AMPK kinases are key molecules (41). We questioned whether sertraline alone or in combination with erlotinib would regulate mTOR and AMPK in EGFR TKI–resistant cells. Our results showed that sertraline dose-dependently activated AMPK and deactivated mTOR in A549 cells (Figure 5D). When combining with erlotinib, sertraline at lower concentration dramatically strengthened erlotinib in reciprocally regulating AMPK and mTOR (Figure 5E). Activation of mTOR leads to phosphorylation of many target proteins related to translational machinery (42, 43). In our study, ribosomal protein S6 kinase (S6K) was also apparently suppressed by sertraline alone and in combination with erlotinib (Figure 5, D and E).

The kinase mTOR is a component of 2 mTOR complexes, mTORC1 and mTORC2. mTORC1 is a major negative regulator of autophagy. The PI3K/AKT pathway is an upstream major modulator of mTOR (42, 43), and the MEK/ERK pathway is a downstream effector of AMPK and mTOR (44). Both of them are involved in the regulation of the autophagy process. Therefore, we further detected the activities of AKT and ERK in treated cells. Our results showed that sertraline had little effects on the phosphorylation of AKT and ERK, even at a higher concentration of 20 μM (Figure 5, D and E). This result of sertraline on AKT activity was in accord with previous findings that phospho-AKT status was unchanged upon sertraline treatment in MCF-7 cells (45). Although the combined regimen of sertraline and erlotinib significantly suppressed the phosphorylation of AKT and ERK, these effects were largely due to potent inhibition of these signaling molecules by erlotinib alone. The regulatory action of the sertraline-containing treatments on the AMPK/mTOR/S6K signaling pathway was similarly observed in PC9/R cells (Supplemental Figure 6). Together, these results suggest that the AMPK/mTOR/S6K signaling pathway is a potential target of sertraline alone and of the combined treatment.

### AMPK plays a critical role in sertraline-mediated cytotoxicity.

**Figure 6:**
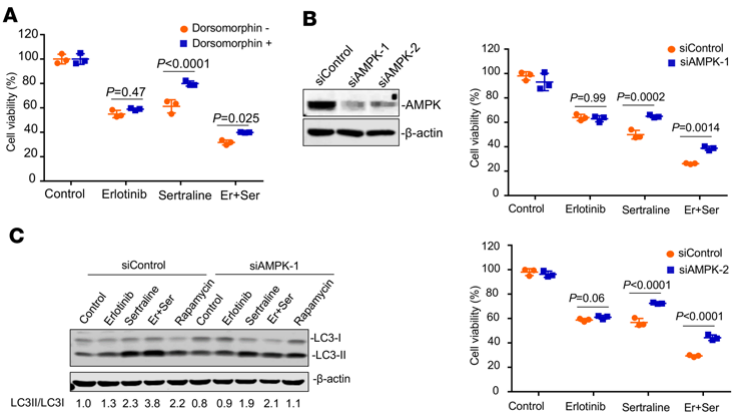
Pharmacological blockade and genetic knockdown of AMPK impaired the effectiveness of sertraline and the drug pair. (**A**) Blockade of AMPK by dorsomorphin significantly inhibited the antitumor activity of sertraline or the drug pair in A549 cells. Cells were pretreated with dorsomorphin (10 μM) for 2 hours, followed by the treatments of erlotinib (10 μM), sertraline (10 μM), or drug combination for 48 hours. (**B**) Silence of AMPK significantly impaired the anticancer effect of sertraline or sertraline plus erlotinib. A549 cells transiently transfected with siControl or siAMPK were treated with erlotinib (10 μM), sertraline (10 μM), or drug combination for 48 hours. Cell viability was determined by the CellTiter-Glo luminescent cell viability assay. (**C**) A549 cells were treated with sertraline (10 μM), erlotinib (10 μM), or drug combination for 24 hours after transfecting with siControl or siAMPK-1. The cell lysates were subjected to sodium dodecyl sulfate polyacrylamide gel electrophoresis, followed by immunoblotting with indicated antibodies. All data in **A** and **B** were represented as mean ± SD (*n* = 3). *P* values were performed by 2-way ANOVA, followed by Sidak’s multiple comparisons test, and *P* < 0.05 was considered statistically significant. Er, erlotinib; Ser, sertraline.

We next examined whether AMPK was markedly involved in the reduced growth of NSCLC cells triggered by the sertraline-containing treatment. We decreased cellular AMPK by using pharmacological inhibitors or siRNAs. Our results showed that pharmacological inhibition of AMPK by dorsomorphin significantly impaired the anticancer effect of sertraline or its combination with erlotinib (Figure 6A). Similarly, siRNA-mediated downregulation of AMPK also significantly reduced the cytotoxic potency of the sertraline-containing treatments (Figure 6B). In addition, the accumulation of LC3-II induced by sertraline or its combination with erlotinib were rescued after siRNA treatment (Figure 6C). Collectively, these data indicate that AMPK plays an important role in the mechanism-of-action of sertraline or the combined treatment in EGFR TKI–resistant NSCLC cells.

### Sertraline enhances the anticancer effect of erlotinib in vivo.

**Figure 7:**
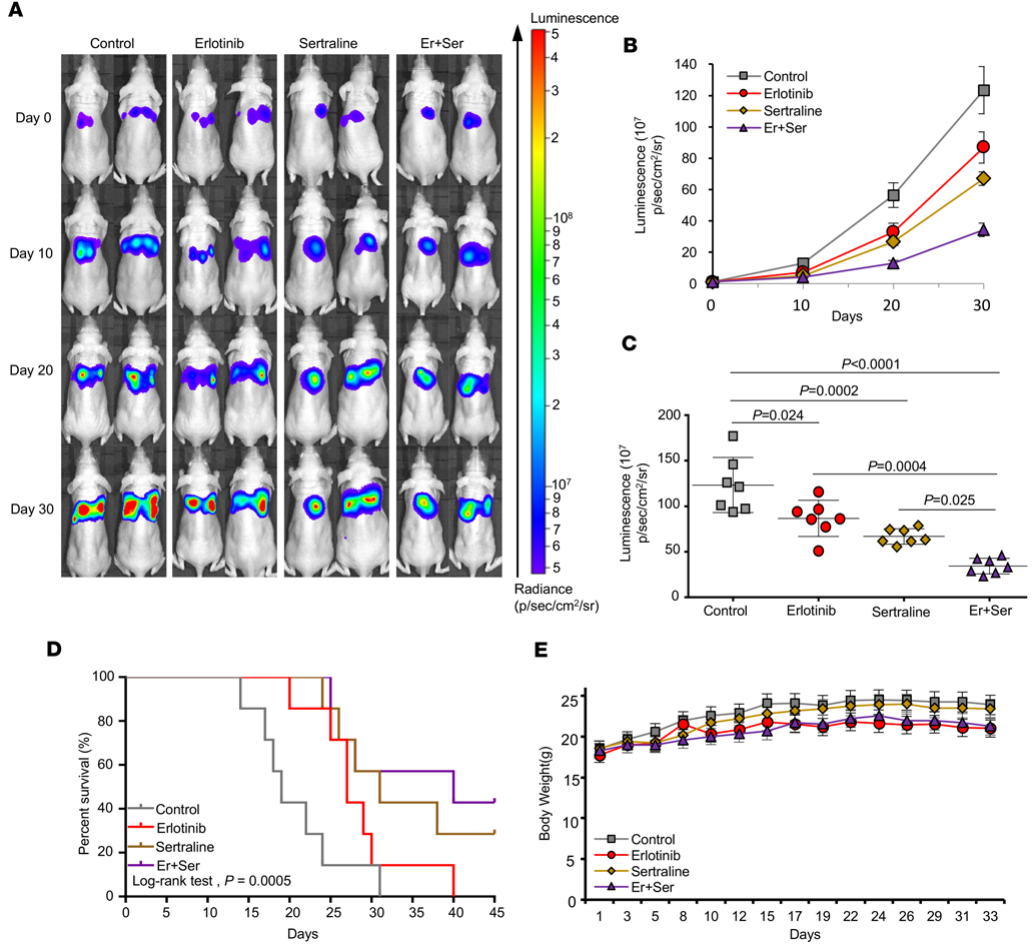
Sertraline enhanced the therapeutic efficacy of erlotinib in an orthotopic NSCLC mouse model. (**A**) Combining sertraline with erlotinib suppressed the growth of EGFR TKI–resistant NSCLC. After being injected with luciferase-labeled A549-luc2 cells, the mice were divided into 4 groups based on the initial bioluminescence (*n* = 7 in each group): the vehicle control (PBS, p.o. daily), erlotinib (50 mg/kg, p.o. daily), sertraline (50 mg/kg, p.o. daily), and erlotinib combined with sertraline (50 mg/kg, p.o. daily; 50 mg/kg, p.o. daily). Bioluminescent images were recorded using a Xenogen IVIS 2000 Biophotonic Imager every 10 days. (**B** and **C**) Quantification of bioluminescence in different treatment groups, as described in **A**. *P* value was analyzed by 1-way ANOVA followed by Tukey’s multiple comparison test. (**D**) The mouse survival curve. *P* value was analyzed by the Log-rank test. (**E**) The body weight in mice. There was no significant difference in mouse body weight between the control group and treated groups. *P* < 0.05 was considered statistically significant. Er, erlotinib; Ser, sertraline.

To investigate the efficacy of the combined therapy in vivo, we established an orthotopic lung tumor model using engineered A549-luc cells. Tumor progression was monitored every 10 days. As shown in Figure 7, A and B, tumors in the vehicle control group grow quickly and diffuse into the whole lung tissue during a 1-month period of treatment. Single-agent treatment of sertraline or erlotinib inhibits the tumor progression at the tested dosage; however, the cotreatment of these 2 drugs exhibits a much more potent antitumor property compared with either single agent alone at the end of treatment (Figure 7C; *P* = 0.0004 vs. erlotinib; *P* = 0.025 vs. sertraline). Notably, the combined therapy dramatically prolonged mouse survival: the control group (*n* = 7; median survival, 19.0 days), sertraline group (*n* = 7; median survival, 31.0 days), erlotinib group (*n* = 7; median survival, 27.0 days), and combination group (*n* = 7; median survival, 40.0 days) (with *P* = 0.0005 when compared with the vehicle control group by Log-rank test; Figure 7D). Note that there was no significant difference in mouse body weight among different treatment groups, suggesting that these treatments were well tolerated in mice at tested dosage (Figure 7E). These results suggest that sertraline potently sensitizes erlotinib to slow tumor progression in vivo.

## Discussion

Lung cancer is the leading cause of cancer-related deaths worldwide in both men and women (46). EGFR-mutant NSCLC was first identified as a distinct and clinically relevant subset of lung cancer in 2004 (47). Although patients with EGFR-mutant lung cancer show initial response to TKIs, the benefits of TKI treatments are gradually weakened due to quick development of resistance by tumors. Thus, there is an urgency to discover new therapeutic agents with novel mechanisms to overcome primary and/or acquired resistance of EGFR TKIs. In this study, we developed a medical genetics–based approach to identify potentially new indications for over 1,000 FDA-approved drugs. Using this computational approach, we found that the antidepressant drug sertraline could sensitize NSCLC cells to erlotinib in vitro and in vivo. Importantly, we showed for the first time to our knowledge that a sertraline and erlotinib combination induced autophagy in NSCLC, as evidenced by their ability to reciprocally regulate the AMPK/mTOR signaling pathway. Collectively, our study reveals the potential role of sertraline in a combined regimen with erlotinib to treat EGFR TKI–resistant NSCLC by the medical genetics–based methodology.

Autophagy is an evolutionarily conserved cellular process that eliminates protein aggregates and dysfunctional organelles in lysosomes. Accumulating evidence has shed light on the importance of autophagy in cancer (48). Previous studies suggested that EGFR was dysregulated in a variety of human cancers and that EGFR TKIs, including erlotinib at clinically relevant concentrations, induced autophagy in NSCLC cells (36). Erlotinib binds to the EGFR ATP binding site, dephosphorylates EGFR, and abolishes the interaction between EGFR and the autophagy protein Beclin 1 (49). These facts inspired us to investigate whether sertraline, either alone or in combination with erlotinib, could suppress tumor growth in NSCLC through the induction of autophagy. Our results systematically showed that sertraline remarkably augmented erlotinib-induced autophagy (Figures 3 and 4). Autophagy is accelerated by AMPK, which is a crucial energy sensor in maintenance of cellular energy homeostasis. At the same time, autophagy is also restrained by mTOR, a basic cell-growth regulator that responds to growth factors and nutrient signals. Both of these intracellular proteins are key regulators of autophagy. Our mechanistic data showed that sertraline not only blocked mTOR phosphorylation, but also suppressed its downstream effector p70S6K. This implied an inhibitory effect on the mTOR signaling pathway mediated by sertraline. Meanwhile, sertraline increased AMPK phosphorylation in a concentration-dependent manner. We hypothesized that the sertraline and erlotinib drug combination promoted autophagy through a reciprocal regulation on AMPK and mTOR in NSCLC cells. Because sertraline targeted the AMPK/mTOR signaling pathway, we investigated the inhibitory activity of sertraline against a panel of 57 kinases. As shown in the Supplemental Table 2, sertraline exhibited weak inhibition on kinases at a concentration of 10 μM, suggesting that sertraline may not be a direct kinase inhibitor. However, other genes or pathways may also be involved in the anticancer activity of sertraline in NSCLC. For instance, it was reported that sertraline could induce ER calcium release in PC3 human prostate cancer cells (50). In addition, sertraline interacted with translationally controlled tumor protein and decreased its cellular levels, resulting in diminished migration properties and colony formation capacity of melanoma cells (51). Therefore, further experiments are needed to clarify whether these genes or pathways contributed to autophagy induction by sertraline.

Sertraline is a selective serotonin reuptake inhibitor, with a high binding affinity toward the serotonin transporter (52). Sertraline is primarily prescribed for depressive disorder, obsessive-compulsive disorder, panic disorder, and social anxiety disorder in adults. It has good tolerability and a favorable safety profile (53). Recently, the antitumor activity of sertraline was identified. Sertraline significantly decreased the expression level of a translationally controlled tumor protein (54). In addition, sertraline potently inhibited AKT phosphorylation and exhibited potential activity against melanoma in vivo (55). In this study, we demonstrated that sertraline enhanced the therapeutic efficacy of erlotinib in NSCLC cells through targeting the AMPK/mTOR signaling pathway.

A previous study has suggested that the peak plasma level of sertraline reached approximately 583 nM at the recommended dosage (200 mg/day) (56) — much lower than what we used in in vitro assays; however, sertraline was able to enrich in lung tissue by nearly 67-fold (approximate 39 µM), allowing appropriate concentration to be achieved to suppress the growth of lung tumors (57–59). The good pharmacokinetic profile of sertraline in lung tissue may relate to its dramatic anticancer activity in vivo. Since our sertraline results were obtained mostly at a concentration of 15 μM at cellular levels, further studies are needed to determine the optimal dosage for its clinical use. In summary, we identified a potentially novel indication of sertraline for NSCLC treatment in several well-known EGFR TKI–resistant cell lines and an orthotopic NSCLC model with resistant xenografts. Given its favorable safety profile (60), the sertraline and erlotinib combination therapy offers a potential therapeutic strategy for NSCLC.

## Methods

### Construction of the DGI network and the GDN.

Three public databases, DrugBank (v3.0) (23), TTD (24), and PharmGKB (25), were used to build DGIs. Drugs were grouped using ATC classification system codes and annotated using MeSH and UMLS vocabularies (27). All drug target–encoding genes were mapped and annotated using the Entrez IDs and official gene symbols based on the NCBI database (26). Four public databases, the OMIM (December 2012) (28), HuGE Navigator (29), PharmGKB (25), and CTD (30), were used to collect GDAs. The OMIM contained 4,132 GDA pairs connecting 2,716 disease genes in 3,294 Mendelian diseases or disorders (December 2012). The HuGE Navigator database includes more than 300,000 literature-curated GDA pairs from more than 30,000 articles. The CTD contains more than 10,000 experimentally validated or literature-curated GDA pairs. In this study, all genes were annotated using Entrez IDs and official gene symbols based on the NCBI database (26). All disease terms were annotated using MeSH vocabularies (26). For each drug-disease–association pair, we counted the number of genes that were associated with a given disease and/or bound by a specific drug. The *P* value was calculated using the Fisher’s exact test, and the *q* value statistical package was used to compute the tail-based FDR for each drug-disease pair via the Benjamini-Hochberg method (31). A cut-off *q* < 0.05 was used to define significantly predicted drug-disease association pairs. All statistical analyses was performed using the R platform (v3.01, http://www.r-project.org/). We further collected a benchmark drug-disease association dataset from the PharmGKB database (25) to evaluate the performance of our statistical model. The ROC curve was used to examine the model performance (61).

### Reagents.

Antibodies against mTOR (7C10) rabbit mAb (catalog 2983), phospho-mTOR (Ser2448) antibody (catalog 2971), p70 S6K antibody (catalog 9202), phospho-p70 S6K (Thr389) antibody (catalog 9205), AMPKα (D5A2) rabbit mAb (catalog 5831), phospho-AMPKα (Thr172) (40H9) rabbit mAb (catalog 2535), Atg5 (D5F5U) rabbit mAb (catalog 12994), Beclin 1 (D40C5) rabbit mAb (catalog 3495), SQSTM1/p62 antibody (catalog 5114), Akt antibody (catalog 9272), phospho-Akt (Ser473) antibody (catalog 9271), p44/42 MAPK (Erk1/2) antibody (catalog 9102), phospho-p44/42 MAPK (Erk1/2) (Thr202/Tyr204) (197G2) rabbit mAb (catalog 4377), and LC3A/B (D3U4C) XP rabbit mAb (catalog 12741) were obtained from Cell Signaling Technologies. Antibody against PARP (catalog CY6850) was obtained from Abways Technology. Antibody against β-actin (catalog A5441) was from MilliporeSigma. Sertraline (catalog S6319), fluphenazine (catalog PHR1792), erlotinib (catalog CDS022564), chloroquine phosphate (catalog PHR1258), avertin (catalog T48402, 152463), and dimethyl sulfoxide (catalog D2650) were purchased from MilliporeSigma. SCH772984 (catalog HY-50846), rapamycin (catalog HY-10219), dorsomorphin (catalog HY-13418A), and bafilomycin A1 (catalog HY-100558) were obtained from MedChemExpress, and 3-MA (catalog S2767) and Z-VAD-FMK (catalog S7023) were obtained from Selleck. All compounds were dissolved in dimethyl sulfoxide.

### Cell culture.

NSCLC cell lines A549, H522, H1975, and PC9 were obtained from the American Type Culture Collection. A fluorescent protein mCherry-labeled subline (A549-mCherry) of the human lung adenocarcinoma cell line A549 was established using a pLVX-mCherry (catalog 632562) lentiviral system with puromycin resistance (Clontech Laboratories). Similarly, A549-luc2 was established using pLVX-mCherry lentiviral vector in which mCherry is replaced by firefly luciferase 2 (GenBank DQ188837.1) and selected with puromycin. The erlotinib-resistant NSCLC cell line PC9/R was generated by gradually exposing parental erlotinib-sensitive PC9 cells to increasing concentrations of erlotinib for a period of 6 months. A549, A549-mCherry, A549-luc2, H522, H1975, PC9, and PC9/R were cultured in RPMI 1640 Medium supplemented with 10% FBS (HyClone Laboratories), 100 unit/ml penicillin, and 100 μg/ml (Thermo Fisher Scientific, catalog 15240062). All cells were incubated at 37°C in a humidified incubator with 5% CO_2_.

### Cell viability assay.

Cancer cells (2 × 10^3^ cells/well) were directly treated with indicated drug concentrations for 72 hours. To determine cell viability, we used a CellTiter 96 AQueous One Solution Cell Proliferation kit (Promega) and a Flexstation III microplate reader (Molecular Devices). The CI of drug combinations with fixed drug ratios was calculated using Chou-Talalay methods by CalcuSyn software (version 3, Biosoft; ref. 34). CI values of <1, =1, and >1 indicate synergism, additive, and antagonism, respectively.

### Apoptosis and cell cycle analysis.

Cells were seeded in 6-well plates and incubated with the indicated drugs for 48 hours. The apoptosis and cell cycle assays were carried out using the BD Pharmingen apoptosis detection kit (BD Biosciences) according to the manufacturer’s instructions and analyzed by flow cytometry (FACSCalibur; BD Biosciences).

### 3-D colony formation assay.

The 3-D colony formation assay was performed as described previously (62). Briefly, A549-mCherry cells were embedded in 0.4% BD Bacto Agar (BD Biosciences) in RPMI 1640 (10% FBS), followed by the treatment with sertraline, erlotinib, and a combined treatment. Fresh culture medium was changed every 3 days. Colonies were counted and photographed with an inverted fluorescence microscope (Olympus) every week.

### Fluorescence analysis of EGFP-LC3 and mRFP-EGFP-LC3 expression.

Cells were transiently transfected with EGFP-tagged LC3 expression construct (pEGFP-LC3) alone or an mRFP/EGFP fluorescent protein tandem-tagged probe (mRFP-EGFP-LC3) (bioWORLD) using lipofectamine 2000 according to the manufacturer’s instruction (Invitrogen). Subsequently, transfected cells were treated with the indicated drugs. After 12 hours, the cells were fixed with 4% paraformaldehyde and observed under a Laser Scanning Confocal Microscope (Leica TCS SP5, Leica Microsystems). To quantify autophagic cells, we counted the cells with 10 or more GFP-LC3 puncta under an inverted fluorescence microscope (IX-71,Olympus). At least 100 cells were counted in each treatment group.

### Transmission electron microscopy.

After being treated with indicated drugs for 24 hours, cells were harvested and fixed following a standard protocol (63). Cells were sectioned at 50 nm thicknesses, and the samples were then stained by 3% uranyl acetate. Autophagosome was examined by a transmission electron microscopy (TEM).

### Western blotting.

Treated cells were washed twice with ice-cold PBS and then lysed with radioimmunoprecipitation lysis buffer (150 mM NaCl, 1 mM EDTA, 100 mM Tris-HCl, 1% Triton X-100, 1% sodium deoxycholate, and 0.1% SDS) supplemented with protease and phosphatase inhibitors (Roche Diagnostics). Proteins were subjected to 12% SDS-polyacrylamide gel electrophoresis and transferred to nitrocellulose membranes. Membranes were incubated with indicated primary antibodies overnight at 4°C, followed by exposure to specific secondary antibodies IRDye 800 (catalog 926-32210; LI-COR Biosciences) or IRDye 680 (catalog 926-68071; LI-COR Biosciences). Protein concentration was determined by bicinchoninic acid assay (Thermo Fisher Scientific).

### Gene knockdown.

The target siRNA sequences against *ATG5* (siATG5-1, 5′-AUCUGAGCUAUCCAGACAA-3′; siATG5-2. 5′-GACGUUGGUAACUGACAAA-3′), *PRKAA1* (siAMPK-1, 5′-GGAUCCAUCAUAUAGUUCATT-3′; siAMPK-2, 5′-AUGAUGUCAGAUGGUGAAUTT-3′), and the scrambled siRNA (siControl) were synthesized by Gima Company. Plasmid pGenesil-1-*Beclin 1* shRNA was purchased from bioWORLD. The target sequence was as follows: shBeclin 1-1, 5′-GGTCTAAGACGTCCAACAACA-3′; shBeclin 1-2, 5′-GCTCAGTATCAGAGAGAATAC-3′. Cells were transfected with 25 nmol/l siATG5 or 3 μg pGenesil-1-*Beclin 1* shRNA plasmid using lipofectamine 2000. The transfected cells were used for experiments after 24 hours. Protein knockdown of ATG5 and Beclin 1 was confirmed by a Western blot.

### A bioluminescent orthotopic NSCLC mouse model.

Six- to 8-week-old male BALB/cA nude mice were obtained from the National Rodent Laboratory Animal Resources. The bioluminescent orthotopic NSCLC mouse model was constructed as described previously (64). Mice were anesthetized with avertin (250 mg/kg). A 5-mm skin incision overlying the left chest wall was made, and the right lung was exposed. Luc2-labeled A549 (A549-luc2) cells (1 × 10^6^) in 100 μl of FBS-free medium were injected into the right lungs of the mice. After injection, the wound was stitched and the mice were observed until fully recovered. Subsequently, the mice were randomized into 4 groups (*n* = 7 in each group): the vehicle control (PBS, per os [p.o.] daily), sertraline (50 mg/kg, p.o. daily), erlotinib (50 mg/kg, p.o. daily), or dual treatments of sertraline and erlotinib. The mouse body weight was measured every 3 days. To observe the orthotopic lung tumors, mice were anesthetized and i.p. injected with D-Luciferin (2 mg per mouse; Promega). Bioluminescence intensity was recorded every 10 days by a Xenogen IVIS-200 Optical in vivo imaging system (PerkinElmer).

### Statistics.

Data was shown as mean ± SD. Data sets consisting of more than 2 groups were analyzed by 1-way or 2-way ANOVA multiple comparison test (set at 5%). Statistical significance was defined as *P* < 0.05. Statistical analysis for survival curve was performed using the Log-rank test by GraphPad Prism software. For medical genetics–based analysis, *P* values were calculated using the Fisher’s exact test and then adjusted for multiple testing (*q* values) for each drug-disease pair using the Benjamini-Hochberg method (31).

### Study approval.

All in vivo experiments with mice were approved by the IACUC guidelines and under an institutional protocol approved by East China Normal University with respect to animal care and welfare assurance (AR2013/06002).

## Author contributions

WL, FC, ZZ, and XP conceived and designed the study. XJ, WL, XS, and FC carried out experiments and analyzed the data. QW, JL, and ML analyzed data. FC, WL, ZZ, XJ, and XP interpreted the results and wrote the manuscript.

## Supplementary Material

Supplemental data

## Version 1 06/07/2018

Electronic publication
