# Supplementary material for: Repurposing sertraline sensitizes non–small cell lung cancer cells to erlotinib by inducing autophagy
Source: JCI Insight. 2018 Jun 7;3(11):e98921. doi: 10.1172/jci.insight.98921 (PMC6124398; doi:10.1172/jci.insight.98921)
Supplement: Supplemental data [file jciinsight-3-98921-s008.pdf]

SUPPLEMENTARY INFORMATION

1. Supplementary figures

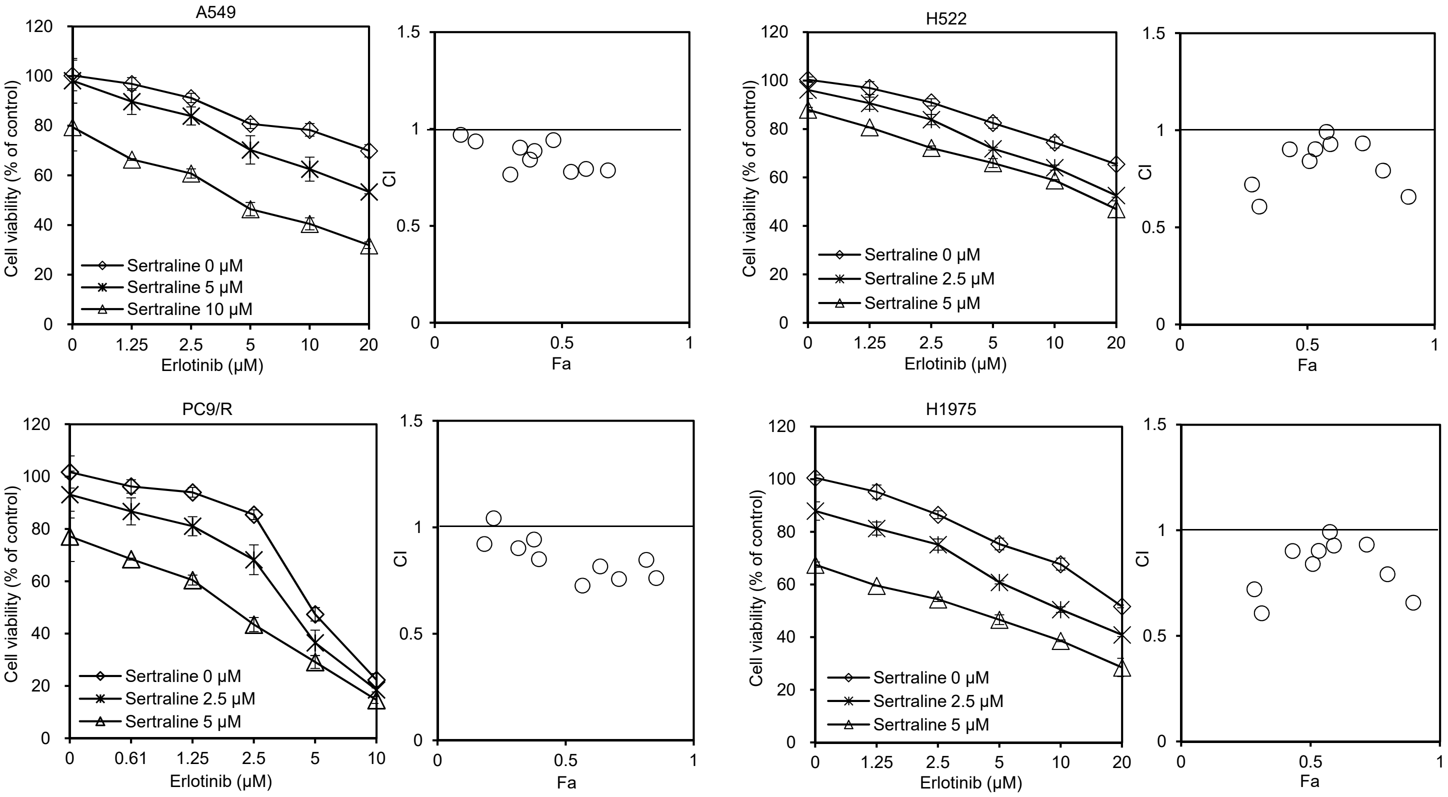

Figure S1. Synergistic effects of sertraline and erlotinib. EGFR TKI-resistant NSCLC cells (A549, H522, PC9/R and H1975) were treated with indicated concentrations of sertraline, erlotinib, and sertraline plus erlotinib for 72 hours. Cell viability was measured by the CellTiter 96® AQueous one solution cell proliferation kit. The combination index (CI) equation as described by Chou-Talalay was generated using CalcuSyn software (Version 2; Biosoft). The data was presented by the Fa-CI plot. The Fa (fraction affected by the dose) and CI value of two drugs at their combination of IC_{50s} were listed in X- and Y-axis. CI values < 1, = 1, and > 1 represent synergism, additive and antagonism, respectively. Data was shown as mean ± SD. All experiments were done independently in triplicate.

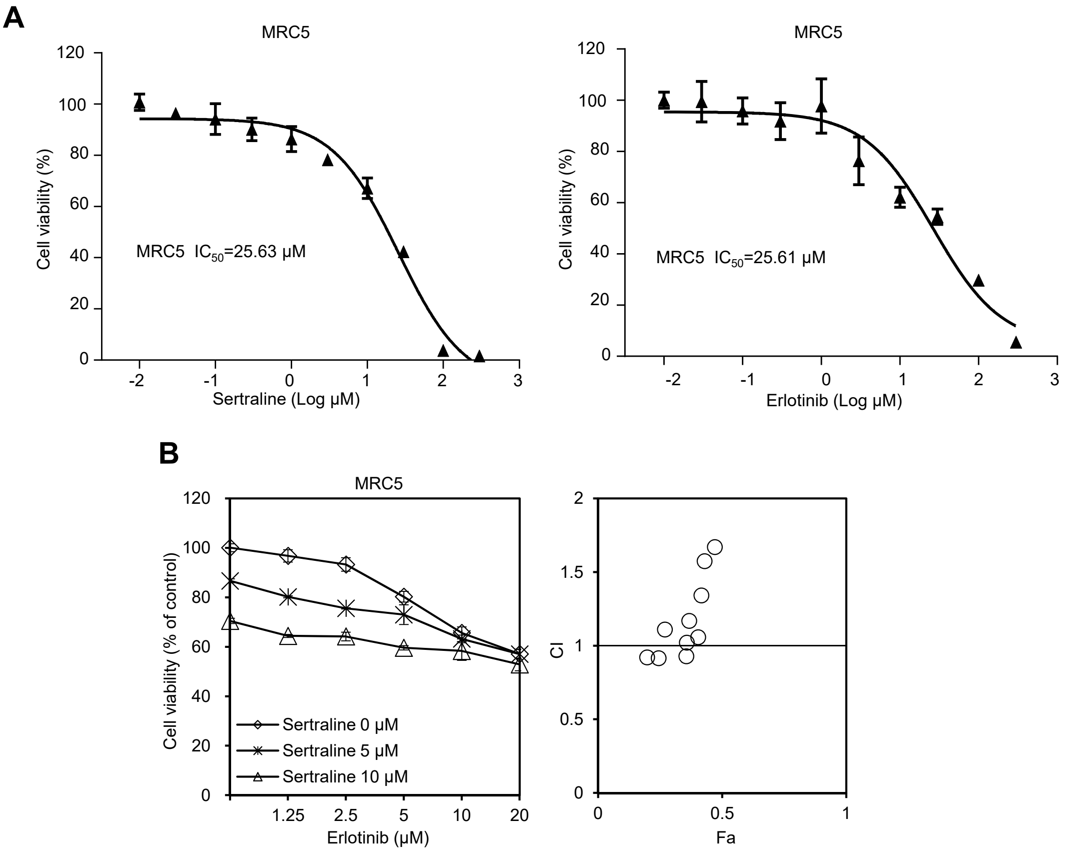

Figure S2. Sertraline and erlotinib show no synergism in untransformed cells. ***A***. The dose-response curves for sertraline in a normal lung cell lines (MRC5). Cells were treated with a series of concentrations of sertraline or erlotinib for 48 hours, and then the cell viability was determined by the CellTiter-Glo® luminescent cell viability assays. ***B***. There is little synergy of sertraline and erlotinib in MRC5 cells. The data was shown in the Fa-CI plots. The Fa and CI value were listed in X- and Y-axis. CI values < 1, = 1, and > 1 represent synergism, additive and antagonism, respectively. Data was shown as mean ± SD. All experiments were done independently in triplicate.

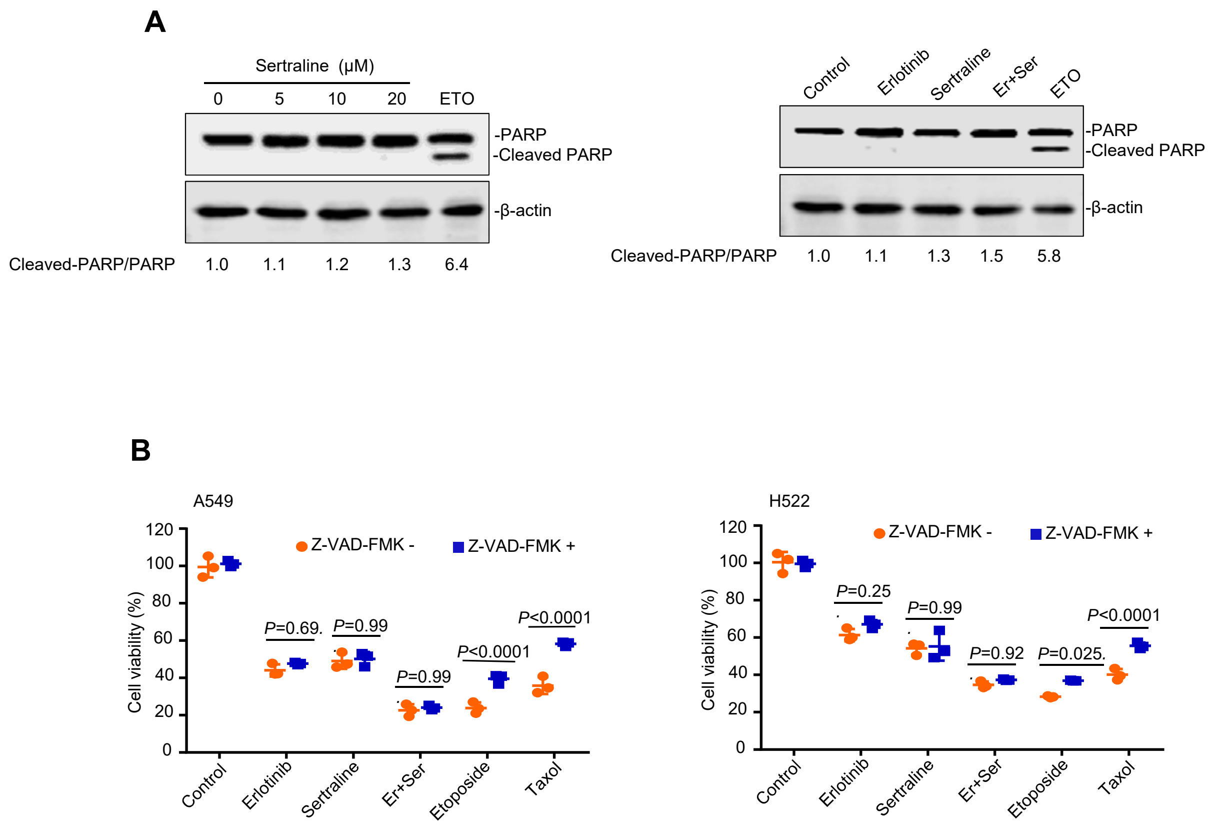

Figure S3. Sertraline-containing treatments do not trigger cell apoptosis. ***A***. Sertraline-containing treatments had little effect on PARP cleavage. A549 and PC9/R cells were treated with sertraline or sertraline plus erlotinib for 24 hours (*n*=3). Etoposide (25 μM) was used as positive control. The PARP expression was probed (*left).* Quantification of relative density of cleaved PARP/PARP was shown (*down*). The density of blots was calculated by the Image J software (NIH; Bethesda, MD). ***B.*** The pan-caspase inhibitor Z-VAD-FMK did not reversed the therapeutic effects of sertraline-containing treatments. A549 and H522 cells were treated with sertraline or sertraline plus erlotinib in the presence or absence of the Z-VAD-FMK (50 μM) for 48 hours. The apoptosis-inducing agents etoposide (25 μM) and taxol (10 nM) were used as positive controls. Cell viability was determined. Data was shown as mean ± SD(*n*=3), and P values were performed by two-way ANOVA followed Sidak's multiple comparisons test. P <0.05 was considered significant. Er: erlotinib, Ser: sertraline, ETO: etoposide.

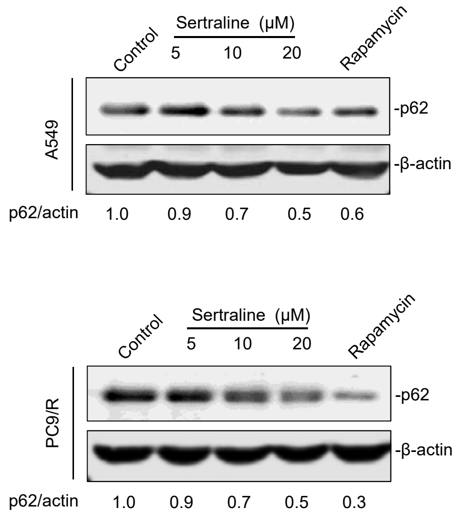

Figure S4. Sertraline decreases the intracellular protein expression of p62 in A549 and PC9/R cells in a concentration-dependent manner. A549 or PC9/R cells were treated with different concentrations of sertraline for 24 hours. Rapamycin was used as positive control. The p62 was probed by the western blotting assay. The representative images (*upon*) and relative were shown (*down*). The density of blots was calculated by the Image J software (NIH; Bethesda, MD). Data was shown as mean ± SD (*n*=3).

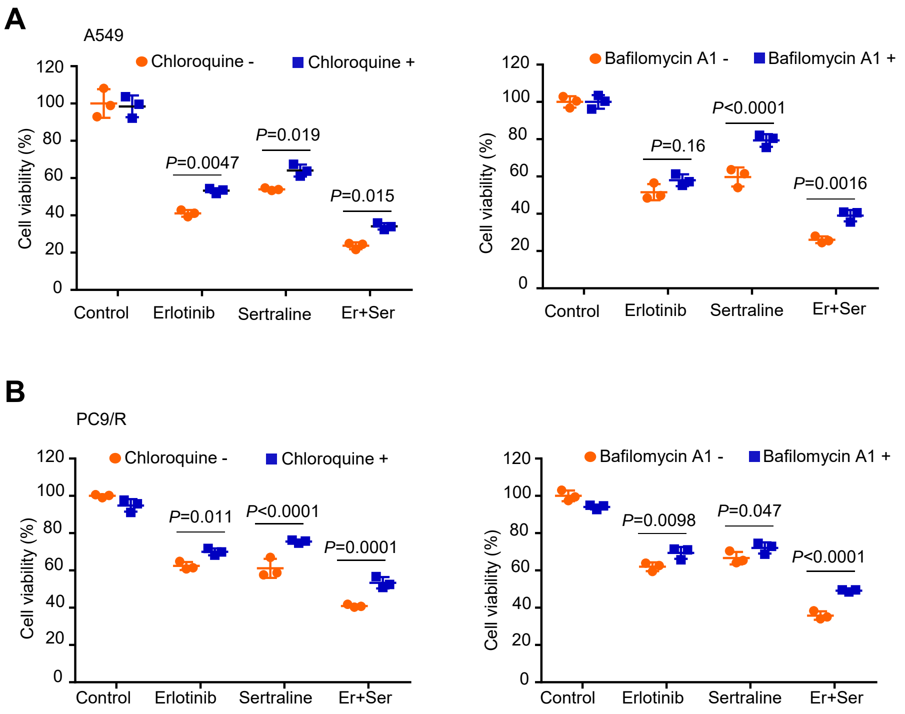

Figure S5. Pharmacological blockade of autophagy by chloroquine or bafilomycin A1 significantly inhibited the antitumor activity of sertraline or the drug pair in (A)A549 and (B) PC9/R cells. Cells were pre-treated chloroquine (50 μM) for 2 hours, or bafilomycin A1 (100 nM) for 2 hours, followed by the treatments of erlotinib (10 μM), sertraline (10 μM) or combination for 48 hours. All data was represented as mean ± SD(n=3). P values were performed by two-way ANOVA followed Sidak's multiple comparisons test. P <0.05 was considered significant. Er: erlotinib, Ser: sertraline.

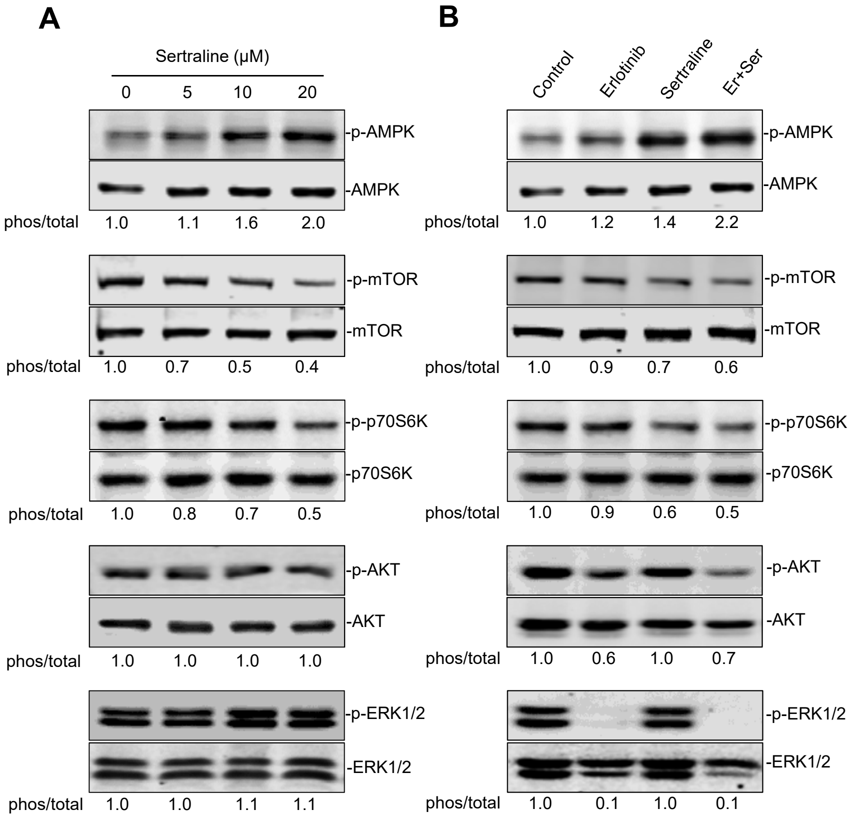

Figure S6. Sertraline alone or in combination with erlotinib induces autophagy through reciprocally regulating the AMPK/mTOR pathway. PC9/R cells were treated with various concentrations of sertraline (5, 10, and 20 μM) or a combination of sertraline (10 μM) and erlotinib (10 μM) for 24 hours. The phosphorylation and basal levels of several key regulators (AMPK, mTOR, p70S6K, AKT and ERK) of autophagy were examined by western blotting. Quantification data of relative density was presented as mean(*n*=3) (*down*). The density of blots was calculated by the Image J software (NIH; Bethesda, MD). Er: erlotinib, Ser: sertraline.

Table S1. Cytotoxicity of sertraline and erlotinib in a panel of NSCLC cell lines.

Cell Line	IC_{50} (μM)	
	Sertraline	Erlotinib
A549	11.08 ± 0.28	25.23 ± 3.39
PC9/R	9.60 ± 1.25	6.80 ± 0.69
H1975	9.40 ± 0.86	49.07 ± 3.62
H522	10.50 ± 1.32	30.76 ± 2.17
PC9	4.40 ± 0.52	0.019 ± 0.00

NSCLC cells (2×10^3 cells/well) were directly treated with sertraline and erotinib for 72 hours, and cell viability was examined. All data was shown as mean ± SD.

Table S2. Inhibitory effect of sertraline on a panel of kinases.

Kinase	Inhibition (%)	Kinase	Inhibition (%)
IRAK1	4.36	p70S6K	29.32
PIM1	9.55	IGF1R	27.25
PDK1	-1.12	CK1δ	2.14
AURA	-11.81	DYRK1α	6.21
HER2	-4.65	mTOR	-9.50
IKKβ	22.53	p38α	4.30
PAK2	12.67	ERK1	23.09
ROCK1	11.26	PKD1	18.21
BRSK1	6.99	MAPKAPK2	13.11
CHK2	-1.11	RSK1	10.21
TSSK1	7.07	CAMK2α	32.11
MARK1	-11.20	DCAMKL2	19.93
CHK1	-5.86	NEK2	10.95
CDK1	-4.51	PKN1	39.20
TAOK2	36.68	PKCδ	33.98
PKCα	34.49	EGFR T790M	-0.80
JAK2	3.17	EGFR(d746-750	-8.64
MST1	27.96	EGFR T790M	-1.15
TNIK	11.70	EGFR	11.47
MAP4K2	14.11	FGFR1	22.92
MASK(MST4	22.91	EphA1	23.88
PI3Kα	7.82	ALK [L1196M]	-26.17
CDK4	-0.67	ALK	19.57
BRAF	-0.22	AMPKα1	6.02
RAF	4.62	GSK3β	-0.99
MET	7.81	FAK	-16.53
SRC	23.44	MEK1	15.81
SGK	31.98	AKT1	33.25
MSK1	13.75		

The inhibitory effects of sertraline (10 μM) on a panel of protein kinases were determined by Shanghai ChemPartner Co., Ltd..
